# A Case of Disseminated Herpes Zoster With Polyneuropathy Following the Third Dose of the Pfizer-BioNTech Vaccine

**DOI:** 10.7759/cureus.33962

**Published:** 2023-01-19

**Authors:** Muhammad Riazuddin, Mona H Jefri, Muhammad I Butt, Hathami I Alsalamah, Aamir Nadeem M Ali Sheikh

**Affiliations:** 1 Medicine, King Faisal Specialist Hospital and Research Centre, Riyadh, SAU

**Keywords:** sars-cov2 vaccines, vaccines, pfizer-biontech, disseminated herpes zoster, varicella zoster reactivation

## Abstract

SARS-CoV-2 pandemic resulted in the fast development of new vaccines, which helped contain the pandemic, but some adverse events started to rise. Recently, post-administration of mRNA-based vaccines, varicella-zoster virus (VZV) reactivation was reported. We report a case of disseminated herpes zoster with polyneuropathy and cerebrospinal fluid (CSF) findings following the Pfizer-BioNTechvaccine. Our observation aims to increase clinicians' awareness of a possible relationship between herpes zoster reactivation and SARS-CoV-2 vaccines.

## Introduction

On March 2020, the World Health Organization (WHO) announced the coronavirus disease 2019 (COVID-19) outbreak as a pandemic [1]. The significant morbidity, mortality, and social and economic impacts have necessitated the development of effective but safe vaccines to prevent and/or decrease both transmission and the disease’s unfortunate outcomes. Although approval for vaccines was granted by most health agencies, adverse reaction profiles have yet to be completely explored. Once a relationship between the vaccine and an adverse event has been observed, a proper investigation should be sought to explain the association. Despite the reported safety in clinical trials [2,3], some side effects are still unknown. Varicella-zoster virus (VZV) reactivation has been described in patients with SARS-CoV-2 infection [4,5] and also after vaccination against hepatitis A, rabies, and influenza, suggesting vaccine-induced immunomodulation [6]. Here we discuss a rare case of disseminated VZV reactivation after four days from the third booster dose of the Pfizer-BioNTech vaccine.

## Case presentation

A 65-year-old gentleman who was in his usual state of health until four days after he received the third booster dose of Pfizer experienced a sudden sharp continuous pain over the right calf muscle, which progressed to the anterior surface of the leg, then ascended in an asymmetrical fashion to the thigh and involved the hip, genitalia, and both lower limbs. The pain was associated with a tingling sensation in a pant-like distribution that is worse over the right lower limb. He sought medical advice, was given painkillers, and was sent home with no improvement. The following day, he developed a rash that started over the posterolateral surface of the right leg as grouped vesicles, then extended to involve different areas of the body, including both lower limbs, back, and head, while sparing the upper limbs and abdomen. There was no headache, weakness, confusion, urine retention, or loss of sphincteric control. He had chicken pox as a child and wasn’t given a booster dose of varicella vaccines. There are no similar conditions in the households or contact with infected individuals.

His past medical history is remarkable for diabetes type II, which is well-controlled on dual oral hypoglycemics with no complications, essential hypertension controlled on a single anti-hypertensive agent, and vitamin B12 deficiency on replacement. He’s generally fit and athletic.

**Figure 1:**
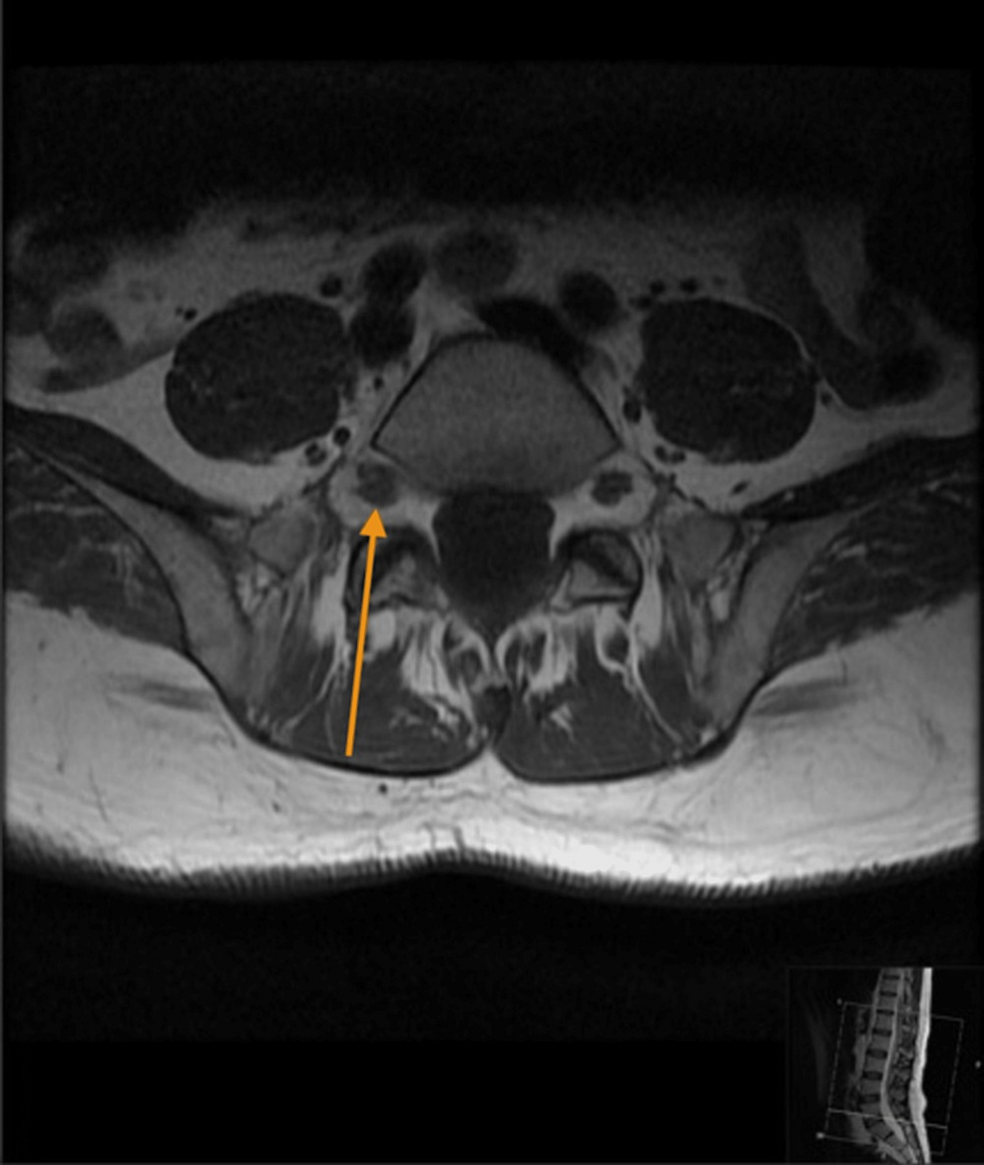
T1 axial without contrast image. Arrow shows the right L5 nerve.

**Figure 2:**
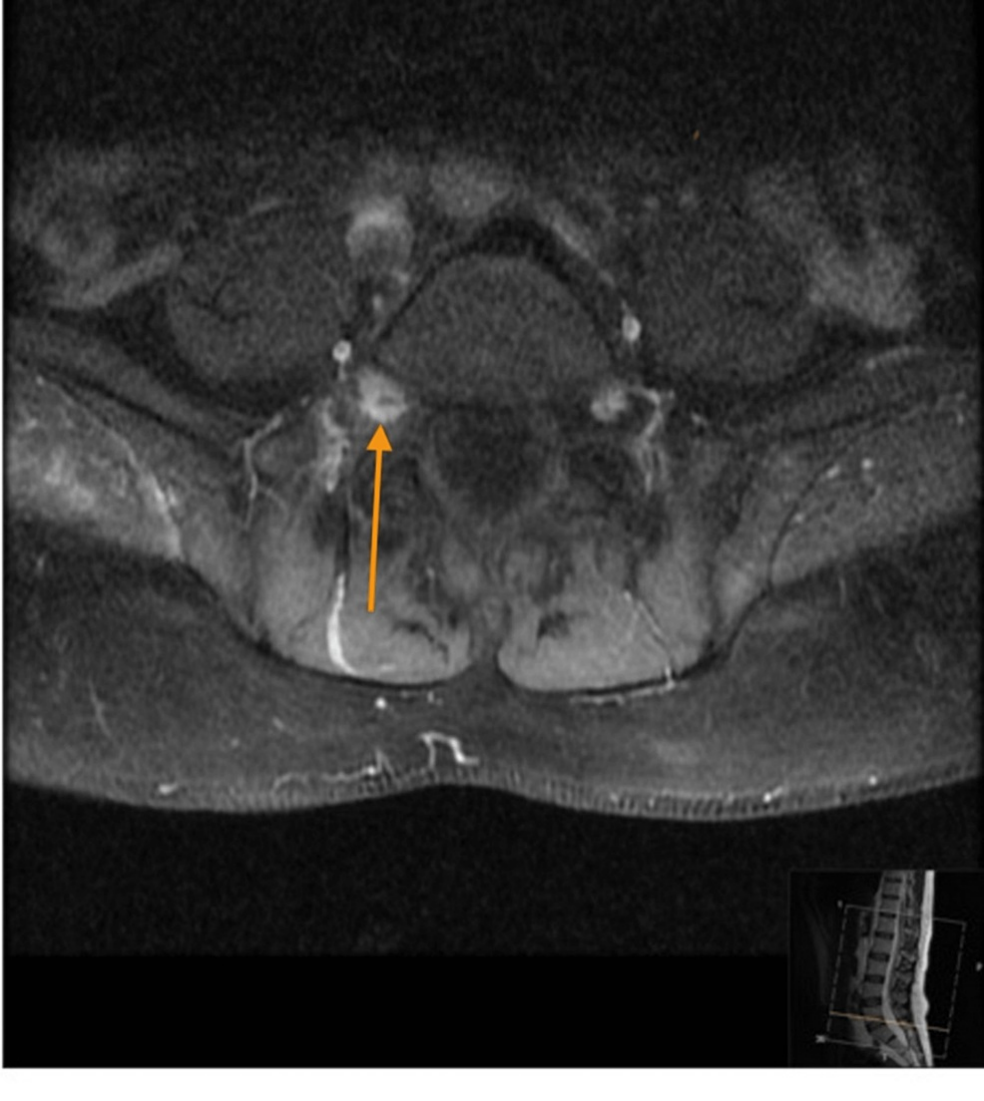
T1 axial fat suppression post-contrast image. Arrow showed prominent enhancement of the right L5 nerve, which is concerning for lumbosacral plexopathy.

Physical examination was significant for a disseminated rash involving multiple dermatomes, some lesions were shiny vesicular in groups, and others were single-spaced vesicles. The neurological assessment revealed impaired right foot dorsiflexion that was noted after the rash, along with decreased pinprick sensation over the dorsum of the foot and lateral surface of the leg. Otherwise, power tone and reflexes were normal all over. The ophthalmological examination was normal. The patient was admitted as a case of disseminated herpes zoster and started on IV acyclovir. Serum varicella zoster IgG and IgM were reactive. The human immunodeficiency virus screen was negative. Magnetic resonance imaging of the spine showed thickening and prominent enhancement of the right L5 nerve, which is concerning for lumbosacral plexopathy. Figure 1 shows a non-contrast T1 axial image with an arrow showing the right L5 nerve. Figure 2 shows the T1 axial fat suppression post-contrast image, and the arrow shows prominent enhancement of the right L5 nerve, which is concerning for lumbosacral plexopathy. Lumbar puncture was done, and cerebrospinal fluid analysis showed a leukocyte count of 500 and a protein count of 1000 with a positive polymerase chain reaction (PCR) for the varicella-zoster virus. A nerve conduction study was carried out and showed the affected common peroneal nerve. He was seen by orthotics and was provided with a supportive aid to improve his foot drop. He was planned to continue 21 days of intravenous acyclovir 15 mg/kg. After two weeks of appropriate management, all vesicles have crusted, and the patient reported improvement in his ability to dorsiflex his right foot.

## Discussion

More than 90 cases of reactivation of VZV following SARS-CoV-2 vaccination were published worldwide [7]. Advancing age has been an important trigger for varicella-zoster virus (VZV) reactivation. More than 50% of the cases is seen in people at 50 years of age or older [8]. This is largely attributed to immunosenescence mechanisms [9]. Other triggers include conditions associated with altered VZV-specific T cell-mediated immunity, including stress, autoimmune diseases, and receiving immunosuppressive therapies [10].

The exact molecular aspects of VZV reactivation and the pathophysiological intersections with SARS-CoV-2 infection or mRNA vaccination remain unclear. It was suggested that SARS-CoV-2-associated lymphopenia and dysfunctional lymphocytes can trigger the reactivation of dominant virus. Vaccines against SARS-CoV-2 can trigger a similar immune response without causing an actual infection; therefore, they would represent a risk factor for VZV reactivation. It is not well understood why mRNA-based vaccines are more implicated. It was speculated that mRNA vaccines may induce a massive cellular shift of CD8^+^ lymphocytes, resulting in a transient impairment of their ability to suppress VZV [7].

A recent gene bioinformatics analysis found that an increased titer of IL-17 was observed in herpes zoster-infected patients [11]. IL-17 is secreted by T helper 17 (Th17) cells, which are differentiated from the naive CD4^+^ T cells. The immune response to the SARS-CoV-2 infection potentiates this differentiation of Th17, resulting in excessive IL-17 production [12]. Yu et al. denoted a possible mediation of IL-17 to the pathophysiology of SARs-CoV-2-related VZV reactivation [13].

## Conclusions

The cases of VZV reactivation after SARS-CoV-2 vaccination is being increasingly reported around the globe. Here, we describe a case of disseminated VZV with CSF findings following the Pfizer-BioNTech vaccine. Establishing a molecular association between both conditions is worth further investigation to guide future preventive and therapeutic strategies.
